# Impact of hemodilution on flow cytometry based measurable residual disease assessment in acute myeloid leukemia

**DOI:** 10.1038/s41375-024-02158-1

**Published:** 2024-01-25

**Authors:** Jesse M. Tettero, Maaike E. Heidinga, Tim R. Mocking, Glenn Fransen, Angèle Kelder, Willemijn J. Scholten, Alexander N. Snel, Lok Lam Ngai, Costa Bachas, Arjan A. van de Loosdrecht, Gert J. Ossenkoppele, David C. de Leeuw, Jacqueline Cloos, Jeroen J. W. M. Janssen

**Affiliations:** 1 Amsterdam UMC location Vrije Universiteit Amsterdam, Department of Hematology, Amsterdam, The Netherlands; 2 Cancer Center Amsterdam, Imaging and Biomarkers, Amsterdam, The Netherlands; 3 Department of Hematology, Radboud University Medical Center, Nijmegen, The Netherlands

**Keywords:** Acute myeloid leukaemia, Acute myeloid leukaemia

## Abstract

Measurable residual disease (MRD) measured in the bone marrow (BM) of acute myeloid leukemia (AML) patients after induction chemotherapy is an established prognostic factor. Hemodilution, stemming from peripheral blood (PB) mixing within BM during aspiration, can yield false-negative MRD results. We prospectively examined hemodilution by measuring MRD in BM aspirates obtained from three consecutive 2 mL pulls, along with PB samples. Our results demonstrated a significant decrease in MRD percentages between the first and second pulls (*P* = 0.025) and between the second and third pulls (*P* = 0.025), highlighting the impact of hemodilution. Initially, 39% of MRD levels (18/46 leukemia-associated immunophenotypes) exceeded the 0.1% cut-off, decreasing to 30% (14/46) in the third pull. Additionally, we assessed the performance of six published methods and parameters for distinguishing BM from PB samples, addressing or compensating for hemodilution. The most promising results relied on the percentages of CD16dim granulocytic population (scarce in BM) and CD117^high^ mast cells (exclusive to BM). Our findings highlight the importance of estimating hemodilution in MRD assessment to qualify MRD results, particularly near the common 0.1% cut-off. To avoid false-negative results by hemodilution, it is essential to collect high-quality BM aspirations and preferably utilizing the initial pull for MRD testing.

## Introduction

Initial induction treatment for acute myeloid leukemia (AML) aims to achieve complete remission (CR), followed by consolidation treatment to prevent relapse [1]. Measurable residual disease (MRD) detection is used to assess a deeper remission status beyond CR and has shown to have additional prognostic value before and after post-induction chemotherapy [2]. MRD can be assessed using different techniques (multiparameter flow cytometry (MFC), quantitative polymerase chain reaction (qPCR), and next-generation sequencing (NGS)), for various diseases (e.g. AML, acute lymphoblastic leukemia (ALL), chronic lymphocytic leukemia (CLL) or multiple myeloma (MM)), using either peripheral blood (PB) or bone marrow (BM), but it is mostly measured in the latter [3–5]. MFC identifies combinations of antigens absent on normal progenitors, referred to as “leukemia-associated immunophenotype” (LAIP), to discriminate between leukemic and normal blasts [6–9]. Standardization and harmonization efforts have been made to ensure reliable MRD results across labs [3, 8, 10–12].

Unfortunately, approximately 30% of MRD-negative patients still experience relapse [13]. Although MRD is not the only determinant of relapse occurrence, a factor that may contribute to false-negative MRD results is hemodilution, caused by the admixing of PB during aspiration of the highly vascular BM [14]. This effect was discovered by retracing radioactively labelled erythrocytes in BM aspirates that were injected intravenously [15, 16]. Hemodilution may lead to an underestimation of the MRD percentage and thus to a false-negative result, due to different proportions of leukemic blasts in PB compared to BM [17, 18]. Consequently, both qPCR and MFC are influenced by hemodilution, making them less reliable when a high volume of PB is aspirated with the BM.

In recognition of this problem, the United States Food and Drug Administration (FDA) advises to take hemodilution into account when assessing MRD and requests that investigators use the first BM pull for MRD assessments [19]. Practical challenges arise due to the required amounts of patient material for the different routine diagnostic tests (flow cytometry, qPCR) that need to be performed, or potential obligations related to sending BM for a clinical trial. Therefore, in practice, it is difficult to adhere to the aforementioned advise to use first pull BM aspirates only and, thus, second or later pulls could result in hemodilution. The European LeukemiaNet (ELN) encourages laboratories to explore strategies for assessing hemodilution, especially when MRD is used for clinical decision-making [3, 10, 20–22].

Various formulas and approaches have been proposed to quantify or compensate for hemodilution in AML and other hematologic diseases [8, 16, 23–28]. These formulas may have additional requirements for laboratory procedures, such as acquisition of paired BM-PB samples or the inclusion of markers such as CD16 that are not typically part of an MRD AML panel. Another way to mitigate hemodilution effects could be by using the primitive marker based MRD assessment (PM-MRD) as a denominator instead of CD45-expressing cells, as this equation is expected to be less influenced by changes in cell proportions [29]. Furthermore, another potential solution is using PB as an alternative specimen to BM. However, despite some smaller studies demonstrating a correlation between the two specimens, the reduced sensitivity of PB-MRD and the lack of validation in large-scale prospective studies make it unlikely that this approach will be the definitive solution in the near future [30–33]. Nevertheless, the exact impact of hemodilution on MRD measurement results remains largely unknown.

In this study, we prospectively assessed the impact of hemodilution by dividing the regular 6 ml of BM into three separate 2 ml pulls, numbered according to their collection order. These three BM samples, along with a PB sample collected on the same day, were individually analyzed and compared at each time point for all patients. Furthermore, we evaluated the sensitivity and specificity of previously established hemodilution formulas by applying them to the four samples of each patient and comparing the outcomes. To facilitate this analysis, a new flow cytometry tube incorporating all necessary antigens, such as the CD16dim population marking granulocytes that are virtually absent in BM and CD117^high^ mast cells were utilized. Finally, we examined the changes in cell populations across subsequent BM pulls and PB to determine the most effective method for distinguishing between the samples.

## Materials and methods

### Patients and treatment

This prospective study included AML patients aged 18 years or older undergoing high-dose chemotherapy following HOVON-SAKK guidelines at the Department of Hematology of Amsterdam University Medical Center (UMC). Patients with acute promyelocytic leukemia (APL) were excluded. Eligible patients in complete remission (CR) at the time of BM aspiration provided written informed consent. BM samples were collected after one or two cycles of chemotherapy. The study adhered to the Declaration of Helsinki (2013) and Medical Research Involving Human Subjects Act (WMO). Additionally, it derives, in part, from the trial registered under the identifier NL9690.

### Dilution series

Standard practice guidelines for BM aspiration were followed [34]. After aspirating 1 mL of BM for inspection of spicules and morphology, three additional tubes were filled (max 3 mL per pull) following a “four eye” principle. The aspiration needle remained stable. PB samples were collected using heparin tubes within three hours of the aspiration. Given that the initial 1 mL of BM was reserved for morphology examination, the first pull dedicated to MRD measurement, referred to as Pull 1 in this manuscript, corresponds to the second pull in the sequential order. Despite aligning with clinical practice, we chose to designate it as Pull 1 for clarity purposes.

### Multiparameter flow cytometry MRD assessment

Before MFC measurement, the white blood cells (WBCs) were counted in all four samples, (pull 1, pull 2, pull 3 and PB). If ≥1,000,000 cells were present, then all samples were stained with the full four-tube eight-color AML-MRD panel, which has been prospectively used in large clinical trials [21, 35]. An additional tube (designated P6) for hemodilution analysis was used, containing monoclonal antibodies against CD10, CD16, CD38 and CD138, which are necessary to validate the previously published formulas for detecting hemodilution. A comprehensive overview of the previously published formulas can be found in Table 1. The panel composition of the four-tube eight-color panel can be found in Supplementary Table S1 and the P6 tube can be found in Supplementary Fig. S1. When an insufficient number of cells was available for the entire panel (four MRD tubes and P6 tube), priority was given to the tube containing the LAIP at diagnosis, followed by the P6 hemodilution tube, and subsequently the remaining tubes in the order of their number. The procedure for measuring MRD was as previously decribed [10, 34, 36]. For previously published hemodilution parameters, the gating strategy utilized in the original publication was replicated where possible. To avoid intra-instrument and inter-operator differences, all samples from one time point were measured on the same FACSCanto II Flow Cytometer (Becton Dickinsons, San Jose, CA, USA) by the same operator. In addition, all samples were gated by the same expert to minimize the inter-gating variability. MRD was determined as the proportion of LAIP-positive cells relative to the total WBC count. Reference samples from both BM and PB, previously measured in other studies, were utilized as control [31]. The blast percentage is calculated based on the cells expressing CD45 and either CD34, CD117, or CD133. This percentage was derived from the LAIP, or if no LAIP is identified, it was determined from the highest among the three markers. A LAIP consists of the CD45 marker, a primitive marker (CD34, CD117, or CD133), and an aberrant marker. In cases where multiple LAIPs where identified within a sample, they are not combined; only the LAIP with the highest quantity was documented. A LAIP percentage exceeding 0.1% of the total WBC was classified as MRD-positive. In parallel with the LAIP method, the Different-from-normal (DfN) approach was employed; nevertheless, none of the samples yielded MRD-positive results using this method [37]. MRD results from the first pull were reported back to the clinic. This study exclusively focused on flow-based MRD measurements, and molecular assays were not concurrently used for MRD assessment in subsequent pulls.

**Table 1:** Overview of previously published formulas to calculate hemodilution.

Reference	Formula for detecting hemodilution	Additional requirements	Validated in this manuscript	Used a cut-off
Holdrinet et al. [16]	Bone marrow purity = [1-(erythrocytes BM/erythrocytes PB) × (leukocytes PB/leukocytes BM)] × 100%	Matched PB	No	No
Delgado et al. [23]	PB contamination index = −3.052 + 0.065 × (%CD10+ neutrophils of granulocytes) −0.609 × (%CD34 + ) − 2.008 × (%plasma cells)	CD10 marker	Yes	Yes
Aldawood et al. [24]	Predicted bone marrow purity = [1 – (Lymphocytes FCM / Lymphocytes PB) × (Leukocytes PB / Leukocytes FCM)] × 100%	PB	Yes	No
Loken et al. [25]	Normalized blast count = (80%/% dim CD16) × blast count	CD16 marker	Yes	No
Schuurhuis et al. [8]	>90% mature neutrophils	CD16 marker	Yes	Yes
Flores-Montero et al. [26]	Suggested blood contamination if mast cell population (CD117^high^) ≤ 0.002%	None	Yes	Yes

### Statistical analyses

A Friedmann test compared percentages of blasts, MRD, and PM-MRD among pulls and PB, followed by Dunn–Bonferroni tests for pairwise comparisons. Differences in outcomes across specimens were assessed using Friedman’s ANOVA test. Wilcoxon tests compared two groups (e.g., pull 1 vs. PB). To determine which population would discriminate best between BM pull 1 and PB, the Chi-squared-test was used. Additionally, the ability of individual features and/or populations to discriminate between BM and PB samples was evaluated in a binary classification task using the area under the receiver operating characteristic curve (ROC-AUC). For every potential threshold, the true and false-positive rate was determined using the scikit-learn package (v1.2.2) in Python (v3.9.10), with the optimal threshold determined as the threshold where the difference between the true and false-positive rate was the smallest. Statistical significance was defined as *p*-value < 0.05. Analyses were performed using GraphPad Prism® Version 5.00, (GraphPad Software, San Diego, CA), R (programming language) with R-package ggplot2 and Python (Python Software Foundation).

## Results

We analyzed 30 patients (median age: 62, range: 19-75), with relevant patient characteristics in Supplementary Table S2. We collected 40 paired BM and PB samples, post-cycle 1 (*n* = 14) and cycle 2 (*n* = 26). Each BM sample had three pulls, totaling 160 samples. All had enough cells for the tube containing the LAIP at diagnosis, except one pull 3 and two PB samples from three patients. Of the 160 included samples, 157 had sufficient cells for diagnosing the LAIP-containing tube. The complete four-tube panel could be measured for 141 samples (88.1%), each with at least 1,000,000 WBC per tube. For 10 samples from three patients, only two tubes were measurable, while for 6 samples from two patients, only three tubes could be measured. Eleven samples had no detectable LAIP above 0.01% MRD in the first pull. Among the 30 samples, we identified 46 distinct LAIPs above the 0.01% MRD threshold in pull 1.

### Consecutive pull analysis

**Fig. 1:**
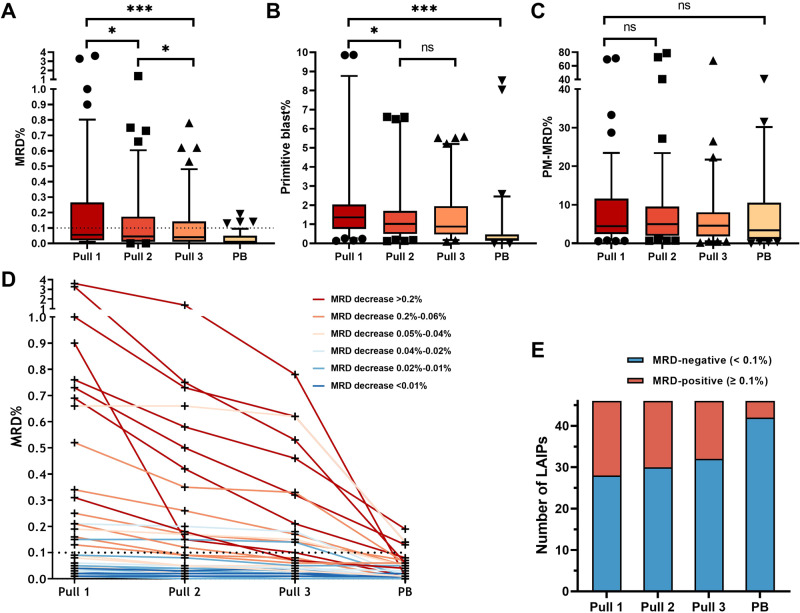
Differences in measurable residual disease (MRD) between samples. **A** Differences in MRD percentages given as percentage of total white blood cell (WBC) count, between pull 1 (first 2 ml bone marrow (BM)), pull 2 (second 2 ml BM), pull 3 (third 2 ml BM) and peripheral blood (PB). Boxes represent the samples between 10%-90% of total. Differences between pull 1 and pull 2 (*p* = 0.025), pull 1 and pull 3 (*p* < 0.001) and pull 2 and pull 3 (*p* = 0.025) were statistically different. All pulls had a significantly higher MRD percentage compared to the paired PB samples. **B** Differences in primitive blasts (CD45+ cells with a primitive marker being CD34+, CD117+ or CD133+) between the three pulls and PB. A significant difference was found between pull 1 and pull 2/pull 3, but not between pull 2 and pull 3. **C** Difference in primitive marker MRD (PM-MRD) depicted as the percentage of LAIP cells with the primitive cells as denominator showed no statistical differences between the pulls and also not between BM and PB. **D** Consecutive MRD results of the individual successive pulls and PB. Colours indicate level of absolute decrease between pull 1 and pull 3. **E** In the 40 paired samples, a total of 46 different leukemia associated immunophenotypes (LAIPs) were identified. Based on the 0.1% cut-off, 18/46 LAIPs (39.1%) were positive in the first pull, compared to 16/46 (34.8%) in pull 2 and 14/46 (30.4%). In the PB samples, only 4/46 (8.7%) of the LAIPs were above the 0.1% cut-off.

We observed significant decreases in the primitive blast- and median MRD% between different pulls and PB samples (Supplementary Table S3). The median MRD% in pull 1 was 0.055%, which was significantly higher compared to pull 2 (0.045%), pull 3 (0.040%), and PB (0.01%) (*P* < 0.001). Pairwise comparisons, adjusted for multiple testing, revealed significant differences between pull 1 and pull 2 (*P* = 0.025), pull 1 and pull 3 (*p* < 0.001), and pull 2 and pull 3 (*P* = 0.025) (Fig. 1A). However, there was no significant difference in the primitive-marker MRD (PM-MRD) levels among the sample pulls and PB (Fig. 1C). Decreases in MRD percentages between consecutive pulls differed among samples (Fig. 1D). Using a 0.1% cut-off, we found that 18 out of 46 leukemia-associated immunophenotypes (LAIPs) (39.1%) were positive in the first pull, compared to 16 out of 46 (34.8%) in pull 2 and 14 out of 46 (30.4%) in pull 3. In PB samples, 4 out of 46 (8.7%) LAIPs were above the 0.1% cut-off, of which all samples were also MRD-positive in BM (Fig. 1E). A sample was considered MRD-positive if at least one LAIP was above the cut-off. Regardless of the decrease in MRD% observed between pull 1 and 2, 9 (22.5%) were considered MRD-positive in both the first and second pulls. One sample became MRD-negative in the third pull (20% MRD-positive or 8 out of 40), and 3 out of 40 PB samples were MRD-positive using the 0.1% cut-off. Based on the data, among the 30 patients, 9 were classified as MRD positive in the first pull. Pull 3 yielded only one “false negative” result. However, when the pulls would be pooled by taking the median of the three pulls, no differences were found compared to the first pull.

### Validation of hemodilution markers

The required P6 tube could only be measured in 28/40 samples because the tube was not available at the start of the study and ten samples had insufficient cell numbers. This tube contained the CD-markers that were not present in our standard four-tube assay but that were necessary to validate the previously published formulas for detecting hemodilution. Of the six formulas, only the one proposed by Holdrinet et al. [16] could not be tested due to the necessity to measure erythrocytes, which are lysed during our regular sample processing steps.

### Peripheral blood contamination index

**Fig. 2:**
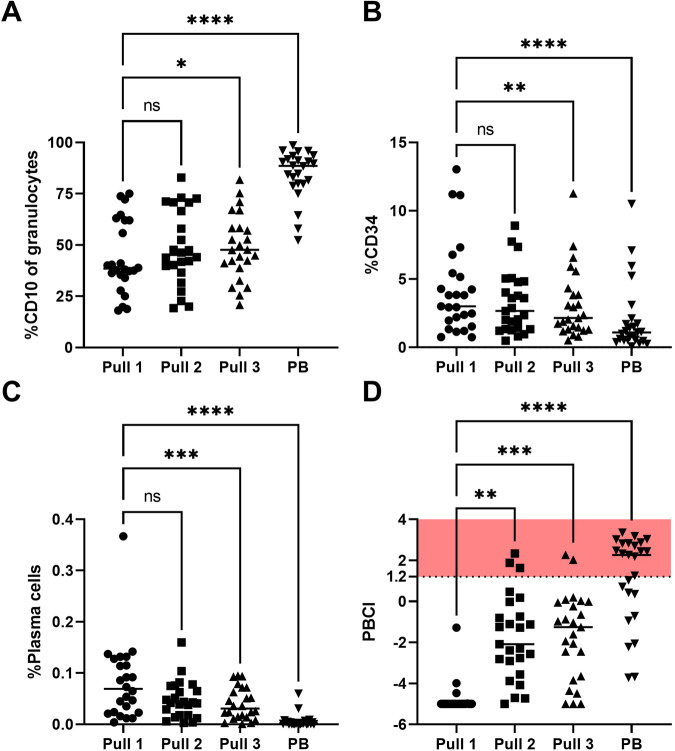
Individual cell populations used to calculate peripheral blood contamination index (PBCI) and PBCI in subsequent samples. **A** CD10+ neutrophils were not statistically different between pull 1 and pull 2, but significantly different between pull 1 and pull 3 and significantly higher in PB. **B** CD34+ population significantly decreased between pull 1 and pull 3/PB. **C** Plasma cells decreased with subsequent pulls, resulting in a statistical significant difference between pull 1 and pull 3/PB, but no difference between pull 1 and pull 2. **D** Calculated PBCI showing significant increase between pull 1 and subsequent pulls. When the 1.2 threshold is applied, three samples from pull 2, two from pull 3 and 15 of the PB samples would be marked as diluted.

The PB contamination index (PBCI) formula consists of three different cell populations, CD10+ neutrophils, CD34+ cells and the CD138+ CD38+ plasma cells (Table 1) [23]. The assumption behind this formula is that CD34+ cells and plasma cells are almost absent in PB, while neutrophils are primarily present in PB. We observed a statistically significant increase in CD10+ granulocytes (Fig. 2A) and a significant decrease in CD34+ cells (Fig. 2B) and plasma cells (Fig. 2C) between pull 1 and pull 3/PB, but not between pull 1 and pull 2. Combining these changes, the PBCI was calculated for all samples, and a significant increase in PBCI was observed from pull 1 to all other samples (Fig. 2D). Applying the published threshold of 1.2 PBCI, which distinguishes contaminated samples from those with good quality, three samples from pull 2, two samples from pull 3. Calculating PBCI in PB showed that only 16/26 PB samples also exceeded this cut-off.

### Predicted bone marrow purity

The formula proposed by Aldawood et al. [24], aimed at determining BM purity, was used to normalize the blast population and not for MRD optimization. Despite this, we applied the formula to our samples as it addresses hemodilution and estimates BM purity. According to the formula, lymphocytes, primarily derived from PB, can be used as a surrogate to estimate pure BM proportions. Analysis of the lymphocyte population in the three BM pulls and PB revealed a significant increase in lymphocytes between pull 1 and PB, but not between the other pulls (Supplementary Fig. S2A). When assessing BM purity for all samples according to the Aldawood formula, a modest but progressive reduction was observed from pull 1 to pull 3 but this did not reach statistical significance (Supplementary Fig. S2B; *P* = 0.20).

### Normalized blast count

The normalized blast count (NBC) formula, originally designed to evaluate and correct blast counts, was used to correct for an estimated general degree of hemodilution, based on a comparison of the proportion of mature myeloid cells (designated as CD16dim cells) to immature blast cells [25]. Calculating the NBC showed no significant differences with the original blast counts in all pulls (Supplementary Fig. S3).

#### Mature neutrophils contamination

**Fig. 3:**
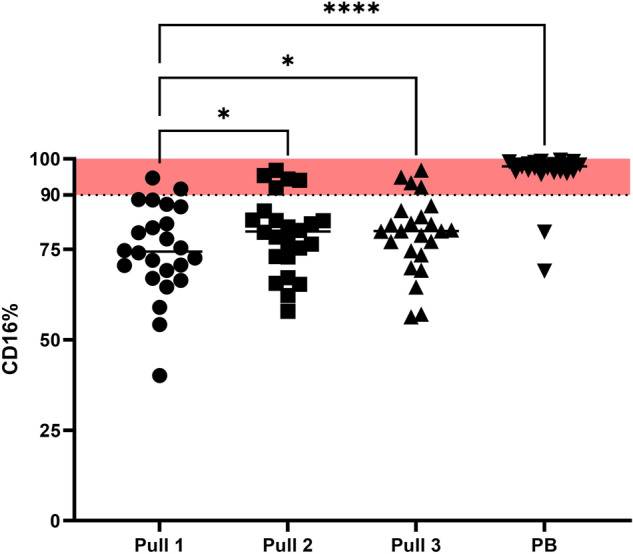
CD16dim expression in successive BM samples and PB. CD16dim cells of the total WBC significantly increased with a median from 74.05% in pull 1, to 79.68% in pull 2 (*p* = 0.030), to 80.02% in pull 3 (*p* = 0.016) and to 97.96% in PB (*p* < 0.001). When the proposed 90% cut-off would be used, two samples from pull 1, 5 samples of pull 2 and 4 samples of pull 3 would be marked as hemodiluted. For comparison, CD16dim expression in PB is shown.

The ELN addressed the issue of hemodilution in their 2018 MRD guidelines [8]. They recommend estimating PB contamination by assessing the percentage of mature neutrophils (CD16dim cells) within the total white blood cell (WBC) population. An increase in the percentage of mature neutrophils >90% would indicate significant hemodilution. We observed a significant increase in the percentage of mature neutrophils with each pull (Fig. 3). The median percentage changed from 74.05% in pull 1 to 79.68% in pull 2 (*P* = 0.030), 80.02% in pull 3 (*P* = 0.016), and 97.96% in PB (*P* < 0.001). Using the proposed cut-off of 90%, two samples from pull 1, five samples from pull 2, and four samples from pull 3 would be identified as hemodiluted. In two PB samples mature neutrophil percentage was <90%.

### Mast cell based blood contamination estimation

**Fig. 4:**
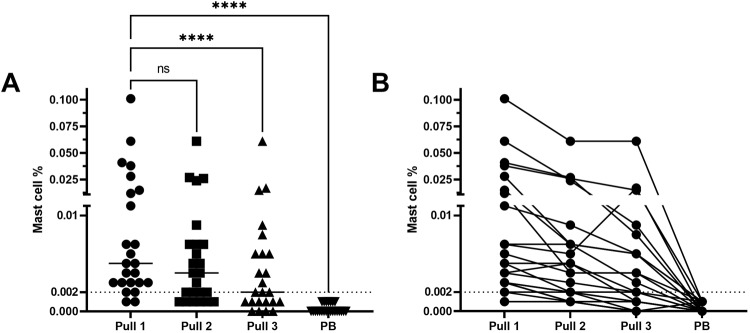
Mast cell population. **A** Mast cell populations (CD117^high^) were measured in all samples, with a decrease between pull 1 and pull 2 (*p* = 0.076), pull 1 and pull 3 (*p* < 0.001) and pull 1 and PB (*p* < 0.001). When the 0.002% cut-off was applied, 4 samples from pull 1, 10 from pull 2, 13 from pull 3 were designated as hemodiluted. All PB samples showed CD117^high^ percentages <0.002%. **B** All samples marked as diluted due to the low mast cell population in the first pull, were also marked as diluted in the subsequent pulls and PB.

**Fig. 5:**
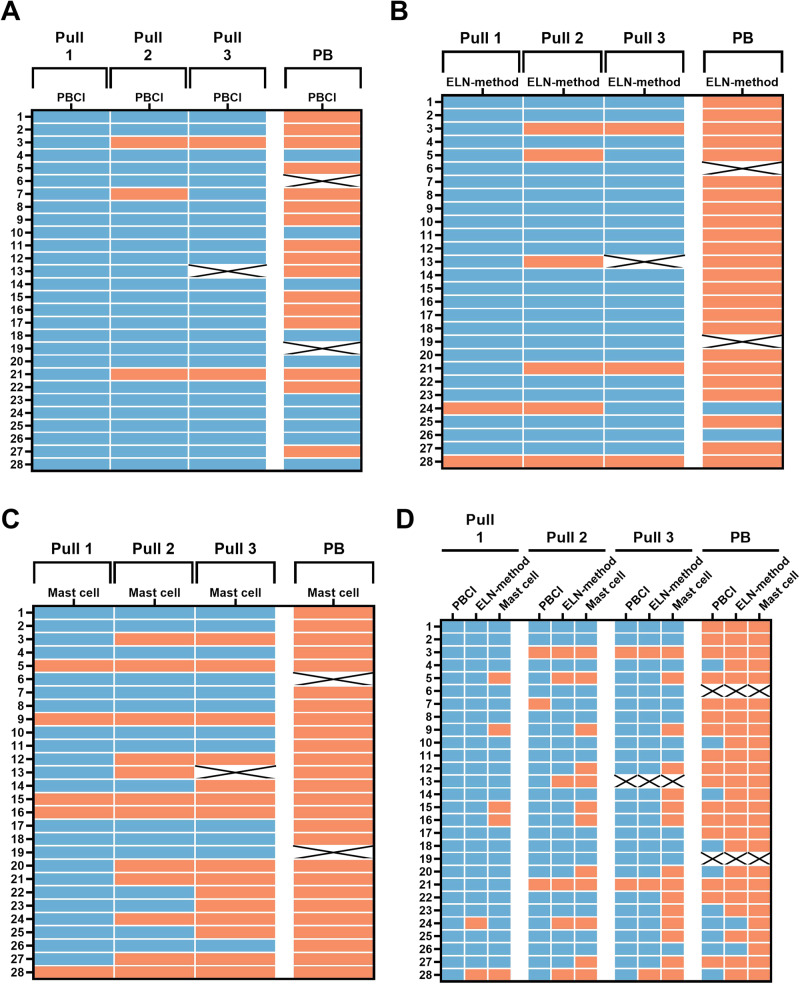
Concordance of samples marked as diluted. **A** Using 1.2 threshold from the peripheral blood contamination index (PBCI), three samples from pull 2 and two from pull 3 were marked as diluted (in red). A sample that could not be measured is shown as X. **B** The European LeukemiaNet (ELN) recommended to mark samples with a CD16dim population of >90% as diluted. With this cut-off, two samples from pull 1, six samples from pull 2, three samples from pull 3 were marked as diluted. **C** A sample with ⩽0.002% mast cells was considered to be diluted. Five pull 1 samples, 12 pull 2 samples and 15 pull 3 samples would meet this criteria. **D** Combination of all three formulas which use a cut-off for assessment of concordance between techniques. Concordance was most profound between the ELN-method (CD16dim population) and the mast cell population, where all samples marked as diluted by the ELN-method except for 1 pull 1 sample, were also marked as diluted based on the mast cell threshold. At all time points, most samples were marked as diluted based on the mast cell population. For comparison, PB results are given in (**A**, **B**, **C**, **D**).

As mast cells (CD117^high^) are solely present in the BM, a decreased percentage can suggest blood contamination (⩽0.002%) [26]. Mast cell populations were measured in all samples, and a decrease was observed between pull 1 and pull 2 (*P* = 0.076), pull 1 and pull 3 (*P* < 0.001), and pull 1 and PB (*P* < 0.001) (Fig. 4A). Applying the 0.002% cut-off, four samples from pull 1, ten from pull 2, and 13 from pull 3 were designated hemodiluted. All PB samples had mast cell levels ⩽ 0.002%. All BM samples with mast cell populations ⩽ 0.002% in pull 1 or pull 2 remained below this limit at subsequent pulls (Figs. 4B, 5C).

### Concordance between methods

By combining the three formulas that use a cut-off level, we assessed the concordance between methods (Fig. 5). Concordance was best between the recommended ELN method, which evaluates CD16dim cells, and the mast cell population method. All samples marked as diluted by the ELN method, except for one pull 1 sample, were also marked as diluted based on the mast cell threshold. The mast cell population method consistently labeled the highest number of samples as diluted in all successive pulls (Fig. 5C).

### Retrospective re-analysis of samples

Among the previously published formulas, only the mast cell formula could be tested retrospectively in previously measured samples since CD117 is a backbone marker in the fixed four-tube panel. We validated mast cells for indicating hemodilution in borderline (0.06-0.09%) MRD-negative samples from HO102 and HO132 trials prospective phase 3 trials (*n* = 18, Supplementary Table S4) [21, 35]. These samples were analyzed after two cycles of chemotherapy to identify potential cases of hemodilution and subsequent relapse. Among these, four samples had mast cell percentages below the threshold, with three patients relapsing within two years, suggesting potential false-negative MRD reports. Furthermore, a sample measured both at the treating center and central lab showed how mast cells could quantify hemodilution (Supplementary Fig. S4). In the treating center, a CD45+ CD13+ CD7+ LAIP comprising 0.18% of WBCs was detected, while the central lab observed the same LAIP at 0.05%, reporting it as MRD-negative. Retrospective analysis of the mast cells percentages showed a level of 0.024% in the MRD-positive sample at the treating center, compared to 0.001% in the MRD-negative sample measured at the central lab facility, indicating hemodilution as the likely cause for the disparity.

### Proposed hemodilution indicator

**Fig. 6:**
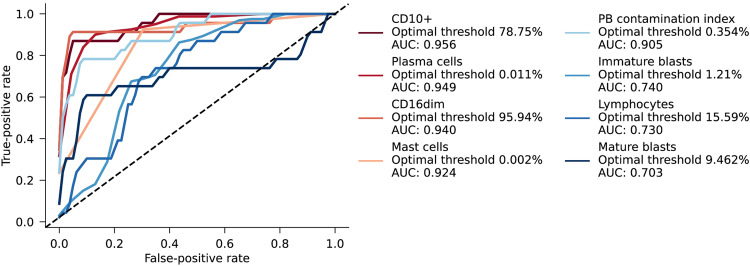
Discrimination of factors between bone marrow (BM) and peripheral blood (PB) based on the receiver operating characteristic curve (ROC). Four parameters (of which the mast cells population is also proposed as formula) and the PB contamination index formula were able to correctly identify BM and PB samples with a AUC above the 0.9. These being, CD10 (AUC: 0.956), plasma cells (AUC: 0.949), CD16dim (AUC: 0.940), mast cells (AUC: 0.924) and PB contamination index (AUC: 0.905).

In addition to the previously published formulas, we assessed various individual cell populations to determine how they changed between the successive pulls and PB. The ability to differentiate between BM and PB samples based on these parameters was evaluated by comparing the Area Under the Curve (AUC) in a Receiver Operating Characteristic (ROC) curve. Four parameters (CD10, plasma cells, CD16dim cells, mast cells, and the PB contamination index) showed AUCs >0.9 (0,956, 0,949, 0,940, 0,924 and 0.905 resp.). Notably, the optimal cut-off for mast cells (0.002%) is the same as the threshold proposed by Flores-Montero et al. [26]. An overview can be found in Fig. 6.

## Discussion

Hemodilution is a crucial factor that poses a significant challenge to reliable MRD assessment, especially near the 0.1% threshold. Despite previous proposals for formulas to detect or quantify hemodilution, there is currently no widely used method or consensus on the standard approach. In our cohort, we observed a significant decrease in MRD percentages between the first 2 ml BM (pull 1) and subsequent pulls, leading to shifts from MRD-positive to MRD-negative status. Although the effect may also be less when the first 6 ml is pooled and this median is closest to our clinical practice to determine the MRD status. This finding is concerning, considering that our study design was relatively conservative, only subdividing the first 6 ml of BM, while the effect might persist in further pulls. Hence, hemodilution prevention or quantification is crucial.

The safest and easiest way to prevent hemodilution is to follow the European LeukemiaNet (ELN) recommendation B3, which suggests taking only 5 mL of BM aspirate from the first pull of the syringe for MRD assessment [3]. However, this option may not be feasible when BM also needs to be collected for different assays such as MFC, qPCR and possibly NGS. Another proposed solution is to reposition or reinsert the needle after the first aspiration, although its impact on MRD results remains uncertain.

Since it is often not possible to prevent hemodilution, the use of formulas to detect or quantify hemodilution appears necessary to warn clinicians for possible unreliable MRD results. However, the formulas we tested have advantages and limitations. The PBCI, relying on CD10, CD38, and CD138 markers not commonly included in MRD assays, showed good discrimination between BM and PB (AUC: 0.905), but this was achieved when using the optimal cut-off in this cohort of 0.354%, which is lower than the proposed 1.2%. When using the proposed 1.2% cut-off, 10/26 PB samples would still not be designated as hemodiluted, thus providing lower sensitivity for hemodilution. The degrading impact of sample aging on plasma cells and their CD138 is noteworthy; however, it was not a concern in this study since all samples were processed within 24 h. Nevertheless, it is important to acknowledge that this factor could potentially impact the reliability of the formula. The CD10+ granulocytes and plasma cell population by themselves seem to have a good AUC of above 0.9, apart from being used in a formula (Fig. 6). Another formula, the normalized blast count formula, which required an additional CD marker (CD16), showed moderate normalization of blast count and may not be sufficient for hemodilution detection. In addition, the lymphocyte and leukocyte compartments were not considered valuable enough for hemodilution detection. The formula based on the percentage of mature neutrophils (CD16dim cells) within the total WBC population, as recommended by the ELN guidelines, performed well in discriminating BM from PB samples [8]. However, in this smaller cohort, the optimal cut-off in this cohort was not at 90% as proposed, but at 95.94%. With both thresholds, only two PB samples would be marked as not diluted and two pull 1 samples would be marked as diluted. Implementing this formula could be a practical way to quantify hemodilution, although the use of CD16 as a marker may not be standard in all panels. The mast cell population, which depends on the CD117 marker (a backbone marker), proved to be the easiest formula to use and showed good performance, with none of the PB samples exhibiting a mast cell concentration above the proposed threshold of 0.002%.

In accordance with the standard protocol, the initial ml of BM was reserved for morphology analysis and assessment of the BM quality. This could potentially account for the characterization of pull 1 samples as diluted and underestimate the effect of hemodilution. Another possibility is that the mast cell test might be overly sensitive, as the first 2 ml pull of BM samples contained insufficient mast cells, as illustrated in Fig. 5. Nevertheless, we recommend implementing the mast cell population as a quick indication of hemodilution. In cases of borderline MRD-negative samples (MRD between 0.07% and 0.1%), the mast cell concentration can provide additional information to determine whether the negative result is most likely truly negative or possibly affected by hemodilution. If hemodilution is suspected, clinicians can be notified that MRD levels may not be reliable and a new BM aspiration should be advised.

Remarkably, the outcomes of PM-MRD analysis exhibited no statistically significant differences across the successive samplings (Fig. 1C). This observation suggests an increased stability of PM-MRD against hemodilution in comparison to the conventional MRD methodology, but further investigation is needed.

There are still several unresolved questions regarding the impact of hemodilution on MRD outcomes. Prior research has indicated similar blast counts between BM aspirations and biopsies in patients with AML, suggesting that malignant cells are not aspirated in higher proportion to non-malignant cells [38, 39]. However, discrepancies in aspiration of different cell types can arise in specific cases involving markers such as LAIPs with CD56, or other adhesion molecules, potentially due to the adhesive properties of malignant cells or their interactions with the BM microenvironment [40–42]. Therefore, some LAIPs may be more susceptible to hemodilution compared to others. Nevertheless, it is imperative to emphasize that the available dataset currently lacks the requisite scale and scope to definitively address this intricate question.

Limitations include a small sample size of only 30 patients, which may limit the generalizability of the findings. Throughout the study design, we took measures to minimize differences between samples, such as a four eyes principle to ensure the right amount was aspirated, using paired samples on the same flow cytometry machine and analyzes were performed by the same lab technician. However, variability can still arise during processing, including pipetting or gating, which may explain some of the observed variability between pulls, particularly when dealing with small differences of 0.01%.

Future studies should prospectively investigate the relevance of the mast cell formula in particular and correlate the results with clinical outcomes to see if correcting for low mast cell percentages can decrease the false-negative MRD results. However, at this point the formulas can only be used to identify hemodilution and not to correct for it. Once validated, these formulas could be implemented as a standard comment on sample quality in MRD reporting. The differences between BM and PB cell populations should serve as the basis for hemodilution detection formulas. Additionally, with the increasing use of automated gating, an automated hemodilution index could be developed and added to the MRD assessment process, as recently proposed by Hoffman et al. [27]. However, caution must be exercised to avoid script-mediated errors when comparing data sets [43]. Furthermore, detecting hemodilution may also be important in other hematological diseases such as acute lymphoblastic leukemia (ALL) or multiple myeloma (MM), where MRD assessment from BM is critical and hemodilution may also lead to false-negative results. Therefore, we do advise to validate these formulas in these diseases as well [44].

In conclusion, hemodilution is a concern even after minimal BM aspiration and warrants consideration in MRD assessment. We recommend incorporating a hemodilution formula, focusing on CD16dim or mast cell populations (CD117^high^). Additionally, to emphasize the importance of the first pull for MRD measurement, BM tubes should be numbered in order of aspiration, with a strong advice to send in the first pull to the MRD lab and if this would be impossible, include the tube number in combination with the mast cell percentage in the final MRD report. In cases of uncertainty, advising BM aspiration repetition is prudent, especially in MFC-MRD between 0.07% and 0.09% in later pulls.

### Supplementary information

Supplementary file of Tettero et al.

## Data Availability

The datasets used and/or analyzed during the study are available from the HOVON/SAKK AML Group upon reasonable request to corresponding author. Requests to access the datasets should be directed to Jacqueline Cloos (j.cloos@amsterdamumc.nl).
