# Supplementary material for: Impact of hemodilution on flow cytometry based measurable residual disease assessment in acute myeloid leukemia
Source: Leukemia. 2024 Jan 25;38(3):630–9. doi: 10.1038/s41375-024-02158-1 (PMC10912027; doi:10.1038/s41375-024-02158-1)
Supplement: Supplementary file 1 — Supplementary file of Tettero et al. [file 41375_2024_2158_MOESM1_ESM.docx]

***Tettero et al. Supplementary file***

**Table S1: Characteristics of included patients.**

|  |  | **No. of patients evaluated (%)** |
| --- | --- | --- |
| **Total** |  | 30 (100) |
| **Male sex** |  | 19 (63) |
| **Age (years)** | ≤45 | 6 (20) |
|  | 46-65 | 20 (45) |
|  | >65 | 4 (13) |
| **AML type** | De novo | 19 (63) |
|  | sAML | 8 (27) |
|  | tAML | 3 (10) |
| **ELN 2017 risk classification** | Favorable | 6 (20) |
|  | Intermediate | 12 (40) |
|  | Adverse | 12 (40) |

Abbreviations: AML, acute myeloid leukemia; ELN, European LeukemaNet; sAML, secondary AML; tAML, treatment related AML.

**Table S2: Primitive blast and MRD characteristics per pull and PB.**

| Variable* | BM pull 1 | BM pull 2 | BM pull 3 | PB | *P*-value** |
| --- | --- | --- | --- | --- | --- |
| Primitive blast cells (%) | 1.36 [0.13;20.77] | 1.02 [0.12;6.62] | 0.88 [0.09;5.58] | 0.22 [0.01:8.52] | <0.001 |
| MRD% | 0.055 [0.0;3.61] | 0.045 [0.0;1.37] | 0.040 [0.0;0.78] | 0.01 [0.0:0.19] | <0.001 |
| PM-MRD% | 4.45 [0.56;71.14] | 4.99 [0.68;78.67] | 4.57 [0.19;82.71] | 3.37 [0.0;83.69] | 0.15 |

*Variables are denoted as median [min; max]. **Pull differences were tested with the Friedman test.

**Table S3: Overview of re-analyzed samples based on borderline MRD-negative results.**

| **Number** | **Study** | **MRD(%)** | **Event-free survival after 2 years** | **Mast cells (%)** |
| --- | --- | --- | --- | --- |
| **1** | HO102 | 0.09 | Relapsed | 0.067 |
| **2** | HO132 | 0.09 | Relapsed | 0.002* |
| **3** | HO102 | 0.08 | EFS | 0.034 |
| **4** | HO102 | 0.08 | EFS | 0.011 |
| **5** | HO132 | 0.08 | Relapsed | 0.011 |
| **6** | HO132 | 0.08 | Relapsed | 0.000* |
| **7** | HO132 | 0.07 | EFS | 0.050 |
| **8** | HO132 | 0.07 | EFS | 0.047 |
| **9** | HO132 | 0.07 | Dead | 0.007 |
| **10** | HO132 | 0.07 | Relapsed | 0.001* |
| **11** | HO132 | 0.07 | EFS | 0.003 |
| **12** | HO132 | 0.07 | EFS | 0.001* |
| **13** | HO102 | 0.06 | EFS | 0.042 |
| **14** | HO102 | 0.06 | Relapsed | 0.005 |
| **15** | HO132 | 0.06 | Relapsed | 0.507 |
| **16** | HO132 | 0.06 | Dead | 0.026 |
| **17** | HO132 | 0.06 | EFS | 0.013 |
| **18** | HO132 | 0.06 | EFS | 0.003 |

Abbreviations: EFS, event-free survival; HO, HOVON; MRD, measurable residual disease. * = possibly diluted based on the ⩽0.002% mast cell cut-off.

**Figures**


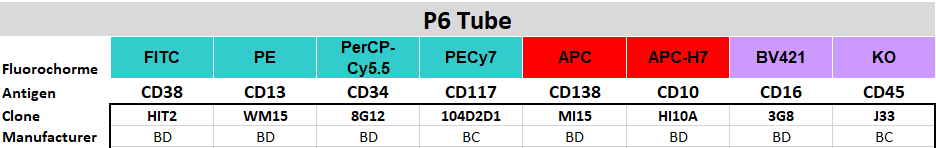


**Supplementary Figure S1. Composition of the P6 Tube**. The P6 tube was specifically designed (labelled as P6) to incorporate additional markers necessary for validating the formulas aimed at detecting hemodilution, which were absent in the standard assay. The supplementary markers incorporated in this tube include CD10, CD16, CD38, and CD138. Markers were made by BD Biosciences (San Jose, CA, USA) or Beckman Coulter (BC) (Brea, CA, USA).


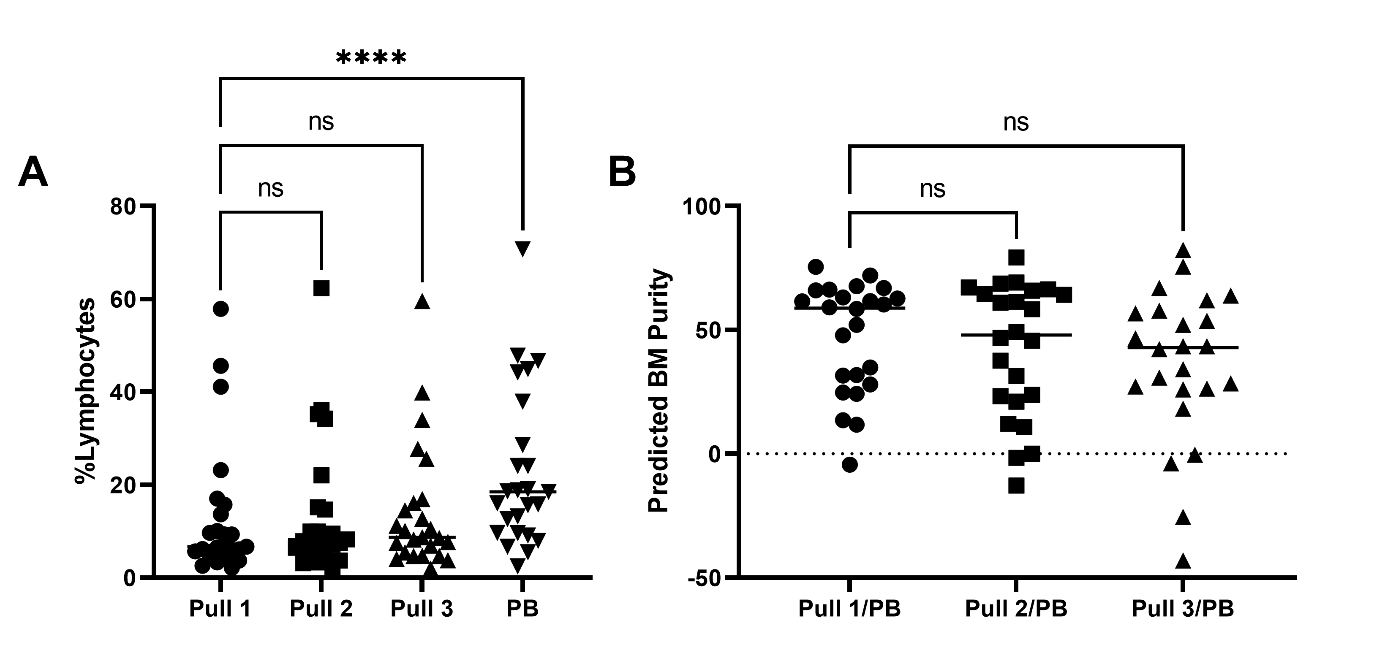
 **Supplementary Figure S2. Predicted bone marrow purity.** (**A**) The bone marrow purity formula is dependent on the increase in lymphocytes, for which a statistical significant difference was found between bone marrow (BM) pull 1 and the peripheral blood samples. However, this increase was not statistically significant between the BM pulls. (**B**) When the predicted bone marrow formulas was used, there was no statistically significant difference found between the purity of pull 1 and subsequent pulls.


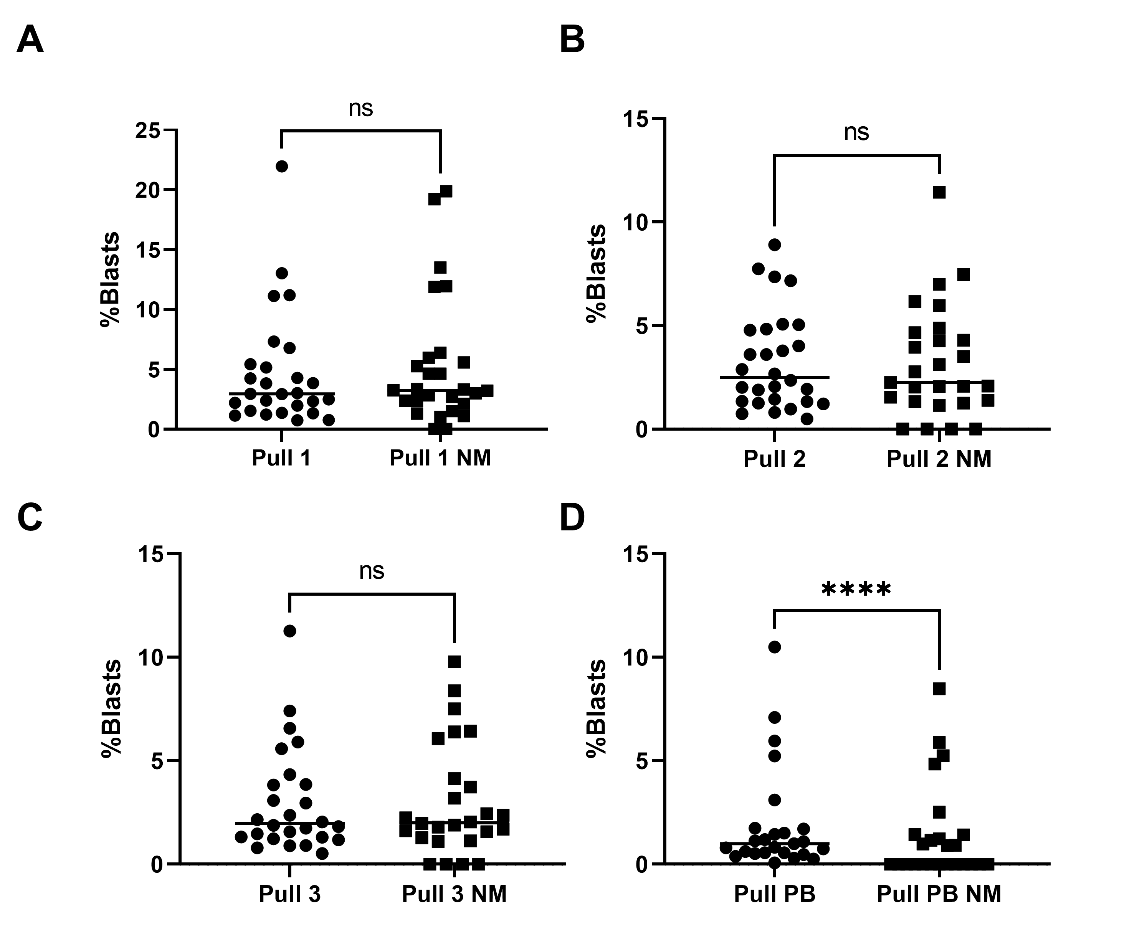


**Supplementary Figure S3. Normalized blast count.** (**A**) The normalized blast count (NM) shows no statistically significant changes in blast percentage in pull 1 between blast count (mean 3.41) and NM blast count (mean 3.23). (**B**) No difference was found between blasts (mean 3.24) compared to NM blast count (mean 2.26) in pull 2. (**C**) Also in pull 3, no significant difference with a mean of 2.37 compared to a mean of 2.01 for the NM blast count. (**D**) The mean for the peripheral blood samples significantly decreased after applying the normalized blast count formulas, with a mean decrease from 1.74 to 0.0001.


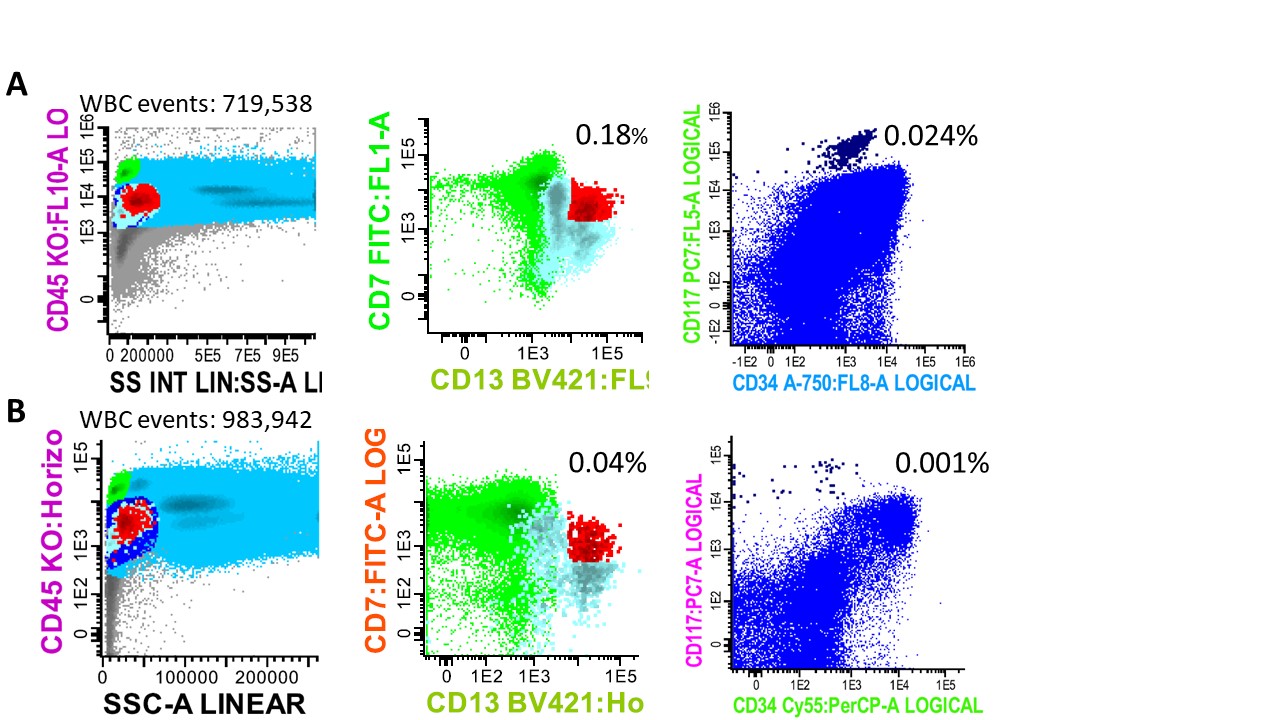


**Supplementary Figure S4. Example of hemodilution and the effect on mast cells**. A sample obtained after two cycles of chemotherapy was evaluated at two different centers, resulting in divergent findings (MRD-positive and MRD-negative) when analyzed by flow cytometry. (**A**) The sample assessed at the center where the bone marrow aspiration was performed exhibited an MRD-positive outcome, with a leukemia-associated immunophenotype (LAIP) of CD45+CD13+CD7+ comprising 0.18% of the white blood cells (shown in red). Upon reanalysis, the mast cell concentration (CD117^hi^, depicted in dark blue) was determined to be 0.024% (i.e. far above the 0.002% threshold). (**B**) Due to participation in a large-scale clinical trial, the bone marrow was sent to a central laboratory for MRD measurement. Despite revealing the exact same LAIP, the percentage was lower, measuring at 0.05%. Consequently, the sample was reported as MRD-negative. To investigate this discrepancy, mast cell percentages were reexamined, which displayed that the population in the central lab sample had decreased to 0.001%, indicative of strong hemodilution as the likely cause for the disparity.
